# Mobile phone applications use while driving in Ukraine: Self-reported frequencies and psychosocial factors underpinning this risky behaviour

**DOI:** 10.1371/journal.pone.0247006

**Published:** 2021-02-17

**Authors:** Tetiana Hill, Amanda N. Stephens, Mark J. M. Sullman

**Affiliations:** 1 Hertfordshire Business School, University of Hertfordshire, Hatfield, United Kingdom; 2 Monash University Accident Research Centre, Monash University, Melbourne, Australia; 3 Department of Social Sciences, School of Humanities and Social Sciences, University of Nicosia, Nicosia, Cyprus; Tongii University, CHINA

## Abstract

Despite the fact that mobile phones have been transformed over the last decade into information and communication hubs that are fundamental to modern life, there is little information on how this has impacted on mobile phone use while driving. The present study was conducted in Ukraine, where this risky behaviour remains a common driving practice, despite legislative bans. A total of 220 (male = 82%; mean age = 35.53; SD = 10.54) drivers completed an online survey assessing frequency of engaging in a range of mobile phone applications while driving. Four variables of the theory of planned behaviour (general attitude and intention towards phone use while driving, social norms towards mobile phone use, perceived behavioural control, the specific beliefs about being able to engage in distracting activities and drive safely), and type A behaviour pattern were also collected. The results showed that, during the last year, 65% of drivers had read a text message and 49% had written a text using mobile phone applications. Likewise, a substantial proportion of the sample reported using social media while driving, by checking (34%), sending or typing a post (25%) on social network applications. Hierarchical stepwise regressions showed that a positive attitude towards mobile phone use while driving and beliefs about being able to drive safely and write or read a text message were significantly associated with the mobile phone applications use while driving. No associations were found between the type A behaviour pattern and mobile phone applications use.

## Introduction

Distracted driving is one of the major risk factors for road traffic injuries and fatalities for drivers, passengers and other road user groups in the world. It often occurs when a driver divides their attention, voluntarily or involuntarily, between driving and an unrelated secondary task. According to the estimates provided by the National Highway Traffic Safety Administration [1], it accounts for approximately 8% of all fatal crashes and 15% of all injury crashes, as well as 14% of all police-reported motor vehicle traffic crashes. Driving distraction may take different forms and vary in terms of the level of interference it can cause, meaning that not all distraction activities have the same impairment on driving performance. Young et al. [2] categorised driver distraction into four distinct types: visual (focusing one’s visual attention on a secondary task), auditory (focusing one’s attention on auditory signals and misfocusing on the road environment), physical (removing one’s hand(s) from the steering wheel to manipulate other objects), and cognitive (thinking about something unrelated to the driving task). Each secondary task may cause one or more types of driver distractions depending on its complexity and demands on driver mental workload [3,4].

There is ongoing debate regarding which secondary tasks have the most detrimental effect on driving performance, however, mobile phone use while driving is considered as one of the most important global road safety issues. This is primarily due to the fact that interactions with mobile phones can encompass all four types of distractions (i.e. visual, auditory, manual, and cognitive). By way of example, the results of the three-year Second Strategic Highway Research Program Naturalistic Driving Study (SHRP 2 NDS), employing a sample of more than 3500 drivers, reported that browsing, handheld dialling and handheld text interactions with a mobile phone are associated with an increased crash risk [5,6].

These findings are also supported by the results of the recent systematic review and meta-analysis of the on-road and naturalistic studies [7,8], as well as epidemiological studies (e.g., [9,10]).

The concern over mobile phone crash risk is increasing, as mobile devices become information and communication hubs often fundamental to modern life. For instance, mobile phone functions are no longer limited to making/receiving calls and writing/sending text messages; smart technology allows engagement in a wide range of functions (e.g., emails, social media, personal data for fitness, banking etc.). Some functions may be beneficial for drivers, such as Global Positioning System (GPS), navigation and real-time traffic updates and these are permitted if the mobile phone is secured in an appropriate holder. However, it is well established that some mobile phone applications may significantly draw attention from driving. This includes both observational and naturalistic studies (e.g., [11–13]). For example, naturalistic driving research conducted on 221 Israeli drivers found that drivers touched their smartphones’ screens at least 1.71 times per minute while driving for different purposes including to use applications [12]. Furthermore, another naturalistic study in Finland tracked the frequencies of use of different mobile phone applications while driving in a sample of 30 drivers. The results showed that drivers most commonly used WhatsApp messaging application, with one instance of use having a median duration of 35 seconds and a median of 8 screen touches [14]. An interesting contrast was the fact that navigation application use had a median duration of 3 screen touches and lasted for 11 seconds. The authors concluded that messaging applications (as opposed to image- or audio-based applications) pose the greatest threat to a driver’s ability to control a vehicle. A similar finding was also reported by McNabb and Gray [15] who found that image-based applications, such as Instagram and Snapchat, had no significant effect on brake reaction time (BRT) and time headway (TH) variability in a driving simulator study. More specifically, the findings showed that scrolling through and reading the updates from a Facebook account considerably increased BRT and TH variability in a sample of 18 drivers in the USA. Similarly, another driving simulator research in Spain found that texting on WhatsApp while driving significantly impaired driving performance for all age groups, and especially among older drivers [12]. Collectively, most of the abovementioned studies conclude that the introduction of mobile phone applications has not only dramatically increased mobile phone use among the general population [16] but has also affected the frequency of mobile phone use among drivers (e.g., [11,12,17]). Consequently, and despite legislation strictly prohibiting handheld mobile phone use in most of the countries, many drivers continue to interact with their phones while driving [3,6].

A number of studies have shown that enforcement and penalties are not effective in reducing the use of mobile phones while driving (e.g., [18,19]). Research in to other dangerous and illegal driving behaviours, such as speeding [20] and drink-driving [21] has found the same. That is, drivers will engage in these behaviours more frequently when they do not feel they will get caught, do not believe there is a risk of crash and when they have friends and family who also engage in the behaviour. This suggests that it is important to understand the attitudes toward the behaviour in order to address the problem.

Previous studies have also examined driver attitudes toward mobile phone use. These have focussed on predicting use based on psychosocial variables, such as attitudes (e.g., [22]), beliefs (e.g., [23]), levels of mobile phone involvement (e.g., [24]), and self-reported frequency of texting and calling behaviours (e.g., [25]). Most of these studies report that the prevalence of mobile phone use while driving is directly related to positive attitudes drivers have towards this behaviour and the perceived benefits their use may have [26].

There is also research that has investigated the attitude-behaviour relationship for driver mobile phone use using the Theory of Planned Behaviour (TPB; [27]). Based on this model, mobile phone use can be predicted by understanding one’s intentions to engage in it, which are predetermined by attitudes (positive / negative evaluation of the target behaviour), subjective norms (perceived approval / disapproval of the target behaviour by significant others), and perceived behavioural control (PBC; perceived ease / difficulty of performing the target behaviour). The TPB has been successfully applied in previous studies to explain texting and calling behaviour while driving (e.g., [22–24,28,29]). In addition, using an extended TPB, which included the additional components of anticipated regret, moral norm, mobile phone involvement, and cognitive capture, Gauld et al. [30] explored university student drivers’ intentions to engage in initiating, monitoring/reading, and responding to social interactive technology on a smartphone. However, none of these have attempted to explore the associations between the TPB variables and the proliferation of mobile phone applications use.

Personality is another factor that can underlie the decision to use a mobile phone while driving. For example, Bianchi and Phillips [31] found that problematic mobile phone use was more prevalent among extraverted drivers as they tend to make more calls while driving. Sween et al. [32] identified that greater emotionality, less conscientiousness, openness to experience and honesty/humility were strongly associated with frequent mobile phone use while driving. No previous research, however, has been carried out to explore the associations between the frequency of mobile phone use while driving and personality traits/types that may explain the tendency to multitask, such as the type A behaviour pattern (TABP). TABP has been recognised as one of the individual variables increasing the risk of road traffic accidents (RTAs; e.g., [33–35]).

TABP was originally conceptualised by two cardiologists, Friedman and Rosenman [36,37], who noted that individuals with heart disease exhibit different behavioural patterns to those with no heart disease. As such, those individuals who have a greater competitive need for achievement, time urgency, aggressiveness, and hostility have overall higher odds of having coronary heart disease. When applied to the driving context, it can be predicted that drivers who exhibit TABP may approach various driving situations with a heightened sense of urgency and impatience, which can be a crucial factor leading to RTAs [38]. For example, West et al. [35] found that TABP was strongly associated with speeding behaviour. The results of a study employing a sample of bus drivers in the USA and India showed that drivers exhibiting TABP reported overall higher traffic accident rates per month, in comparison to those who exhibit the opposite type B behaviour pattern (i.e., these individuals tend to enjoy working steadily and do not experience stress due to a lack of achievements; [39]). Lastly, using a large sample of 11.965 French employees aged 39–52 years, Nabi et al. [33] identified that type A drivers had an increased risk of RTAs. It can be concluded that the implications of TABP for traffic safety may be quite severe, meaning that it is important to investigate whether and how TABP is associated with other safety-critical driving behaviours, such as mobile phone use while driving.

### The present study

The present study was conducted with a sample of Ukrainian drivers, for whom mobile phone use while driving is a prevalent behaviour. The main aim of this study was to explore the frequencies of use of different mobile phone applications while driving and their underpinning psychosocial factors. In particular, we investigated the associations between mobile phone applications use and the prevalence of TABP in the Ukrainian drivers, their beliefs about being able to engage in secondary tasks and drive safely, as well as the four TPB components, such as the general attitude and intention towards phone use while driving, social norms towards mobile phone use, and perceived behavioural control.

## Method

### Participants

The sample consisted of 220 fully licenced drivers in Ukraine. To be eligible to take part in the study, participants were required to hold a current driver’s licence, own a mobile phone, and report driving at least once in the past six months. Most participants were males (82%), aged 19–70 years old (*SD* = 10.54, mean age = 35.53). Participants reported holding a driving licence for an average of 4.01 years (*SD* = 1.17, range: 1–5) and driving approximately 17.02 kilometres per week (*SD* = 18.01, range: 0–150).

### Procedure

Firstly, the survey was translated into Ukrainian by a professional translator and checked for consistency by one of the authors (TH) who is fluent in both languages. Secondly, the survey was hosted online using the Qualtrics web surveying platform. Data were collected via a link sent to the personal e-mails of both staff and students of the National Aviation University in Kyiv, Ukraine, using the existing contacts of one of the authors (TH), who is also an alumna of this University. Approval for this study was granted by the University’s ethics committee. The survey was also advertised on a national forum for Ukrainian motorists. All the study participants were encouraged to pass on the link to eligible friends and family (i.e. those who hold a current driver’s licence, own a mobile phone, and had driven at least once in the past six months). Before completing the online survey, the participants were asked to familiarise themselves with the information sheet outlining that the study was voluntary with no compensation for the participation and that their data would be collected and held confidentially and anonymously.

### Measures

#### Demographic variables

Participants were asked to report their age, sex, marital and work status, educational attainment as well as the number of hours spent driving on average each week. They were also asked to indicate the driving purpose based on the ratio of driving for work versus leisure using a 7- point Likert scale (1 = primarily for work; 7 = primarily for leisure).

#### Frequency of mobile phone applications use while driving

To assess the frequency of mobile phone application use while driving, participants were also asked “How often do you do the following on your mobile phone while driving?”:

- Read a text message using mobile phone applications (e.g., Viber, WhatsApp).
- Send a text message using mobile phone applications (e.g., Viber, WhatsApp).
- Read/look at posts on social networks (e.g., Facebook, VK, Odnoklassniki).
- Send or make posts on social networks (e.g., Facebook, VK, Odnoklassniki).
- Make a video call (e.g., FaceTime, Skype).
- Make a call using mobile phone applications (e.g., Viber, WhatsApp).
- Write an email.
- Read an email.
- Type an address into maps (e.g., Google maps, Yandex).
- Use an intellectual assistant (e.g., Siri, Majel).
- Take photos “selfies”.

Participants were asked to rate each of the options using a 7-point Likert scale (1 = more than once a day; 4 = one or two times a month; 7 = never). The mobile phone applications included in the survey options were selected based on their popularity among the Ukrainian population in 2015 when the study was conducted.

#### Theory of planned behaviour variables

To investigate the associations between the TPB variables and various mobile phone applications use while driving, participants were asked to rate four items extracted from the TPB questionnaire developed by Walsh et al. [29]. Using a 7-point Likert scale (1 = strongly disagree; 7 = strongly agree), participants were asked to indicate their level of agreement with the following statements: In the next week, to what extent do you agree or disagree that:

- It is likely you will use your mobile phone while driving (measuring general intention to use a mobile phone while driving).
- Using your mobile phone while driving will be good (measuring general attitude towards mobile phone use while driving).
- Those people who are important to you would want you to use your mobile phone while driving (measuring social norms towards mobile phone use while driving).
- You have complete control over whether you use your mobile phone while driving (measuring perceived behavioural control).

#### The Bortner Type A scale

This self-report scale was used to assess TABP [40]. It contains 14 items, each consisting of opposing statements placed on a continuum ranging from the extreme TABP to the absence of TABP. Participants were asked to indicate their position between the two extremes using an 11- point Likert scale (e.g., 1 = casual about appointments; 11 = never late). The Bortner Type A scale is a unidimensional scale, the reliability of which in the current sample was acceptable, with Cronbach’s alpha of 0.74.

#### Beliefs about being able to engage in secondary tasks and drive safely

To assess participants’ beliefs about their ability to safely use a mobile phone while driving, four items were added to the survey that were retrieved from a questionnaire developed by White et al. [25]. The questionnaire was developed based on a beliefs-based TPB approach aimed at investigating the direct determinants of intentions to engage in various risky behaviours, such as mobile phone use while driving. Similar to the original questionnaire, in the current study, participants were asked to rate these items (e.g., “I am able to drive safely and send a text message at the same time”, “I am able to drive safely and talk on a handheld phone at the same time”) using a 7-point Likert scale (1 = strongly disagree; 7 = strongly agree).

### Data handling and analysis

All analyses were undertaken using SPSS v.22. There were no missing data. The variable for the ratio for driving was recoded into a binary variable representing driving “primarily for work” (scores of 1 to 3) and “primarily for leisure” (scores of 4 to 7). Mobile phone frequency was recoded as a binary variable for hand-held calls (included making and received calls) and text messaging (included writing or reading text messages) while driving. To explore the associations between the variables Spearman correlations were conducted, which are robust to ordinal data. Cohen’s effect sizes of ≤ .29 as small, between .30 to .49 as medium and ≥ .50 as large, were used to describe the relationships [41]. Hierarchical stepwise regressions were used to explore the psychosocial factors related to different types of mobile phone applications use while driving.

## Results

### Frequency of mobile phone use while driving

Table 1 presents the frequencies of self-reported interactions with the mobile phone applications while driving. The majority of the sample (65%) reported they would read a text message and 49% reported they would type one while driving. Making a call and/or a video call using mobile phone applications were less common amongst the Ukrainian drivers, with nearly 25% of the sample reporting they would make a call using the applications and 21% reporting they would video call someone while driving. No less concerning, a significant percentage of the sample reported engaging in social media while driving: 34% of drivers checked and 25% sent or typed a post on social network applications. Notably, 32% of drivers reported dealing with emails while driving and 38% typed an address into maps applications while driving. Just under half of the participants (41.4%) reported taking selfies while driving at least once a year. The least common behaviour amongst the drivers was using an intellectual assistant (15.5%).

**Table 1:** Self-reported mobile phone applications usage while driving (N = 220).

While driving, how often do you…	More than once a day (%)	Daily (%)	1–2 times per week (%)	1–2 times per month (%)	1–2 times in six months (%)	Once a year (%)	Never (%)
Read a text message using mobile phone applications	15.9	12.3	25.0	5.5	2.7	3.6	35.0
Send a text message using mobile phone applications	7.7	14.5	13.6	7.3	4.1	1.8	50.9
Read or look at posts on social networks	13.2	2.7	4.1	7.3	2.3	4.1	66.4
Send or make posts on social network	12.7	2.3	2.3	3.6	0.9	3.2	75.0
Make a video call	5.0	5.5	4.1	3.6	0.9	1.8	79.1
Make a call using mobile phone applications	10.0	3.2	5.9	2.3	2.3	0.9	75.5
Write an email	10.0	5.0	8.2	3.2	2.7	2.7	68.2
Read an email	9.1	5.9	5.0	3.2	5.0	3.2	68.6
Type an address into maps	8.6	5.5	4.5	8.6	4.1	6.8	61.8
Use an intellectual assistant	8.2	0.9	2.7	1.4	2.3	0.0	84.5
Take photos “selfies”	8.6	7.7	5.5	7.7	3.2	8.6	58.6

### Descriptive variables and intercorrelations between variables

Table 2 displays the correlations between engagement with a mobile phone while driving and age, sex, hours driving per week, TABP, beliefs about being able to drive safely and interact with a mobile phone, as well as the TPB variables. As can be observed from the table, very few relationships emerged between these variables. There were no significant relationships between any type of mobile phone interaction with age, TABP or hours driving per week. Small but significant associations were found between beliefs about being able to drive safely and read or write a text message with reading an email, typing an address into maps and taking “selfie” photos while driving (*r*s ranged from .14 to .23, *p*s < .05). General attitudes towards using a mobile phone while driving and the belief about being able to safely drive and write a text message were also weakly related to reading social media posts and making a call using applications (*r*s ranged from .14 to .18, *p*s < .05). In all instances, drivers with stronger beliefs about their ability to engage in mobile phones use while driving and the benefits of doing so reported more frequent interactions with their mobile phones while driving.

**Table 2:** Associations between engagement with different mobile phone applications while driving and age, sex, hours driven per week, TABP, specific beliefs about being able to engage in secondary tasks and drive safely, and TPB variables (N = 220).

While driving, how often do you…	Age	Sex	Hours driving per week	TABP	Able to drive safely and read a text message	Able to drive safely and write a text message	Able to drive safely and talk on phone	Able to drive safely and talk with handsfree unit	General intention towards mobile phone use while driving	General attitudes towards mobile phone use while driving	Social norms towards mobile phone use while driving	Perceived behavioural control
Read a text message using mobile phone applications	.03	.07	-.18**	.10	-.21**	-.24**	-.13	-.05	-.05	-.09	-.07	.07
Send a text message using mobile phone applications	.02	-.02	-.13	.07	-.22**	-.27**	-.14*	-.05	-.11	-.12	-.14*	.52
Read/look at posts on social networks	.02	-.07	-.04	.01	-.11	-.18**	-.06	.04	.12	-.16*	-.11	-.01
Send or make posts on social network	-.01	-.05	.02	.01	-.11	-.14*	-.04	.04	-.02	-.11	-.07	.01
Make a video call	-.06	-.02	.01	.02	-.06	-.10	-.05	.01	-.05	-.12	-.08	.03
Make a call using mobile phone applications	-.03	-.11	-.02	.02	-.11	-.16**	-.09	-.01	-.08	-.14*	-.09	.06
Write an email	-.05	-.13	-.01	-.01	-.13	-.16*	.01	.07	-.08	-.18**	-.14*	.06
Read an email	-.06	-.12	.01	-.03	-.14*	-.18**	-.01	.07	-.08	-.17*	-.14*	.04
Type an address into maps	.01	-.04	-.06	.01	-.19**	-.22***	-.06	.02	-.10	-.15*	-.13*	.03
Use an intellectual assistant	-.01	-.02	-.03	.01	-.07	-.06	-.01	.01	.03	-.05	-.01	.01
Take photos “selfies”	-.03	-.10	.02	.06	-.20**	-.23***	-.06	-.04	-.03	-.03	-.11	.05
Variable Mean (SD)	35.53 (10.54)	--	17.20 (18.03)	88.78 (17.57)	2.09 (1.56)	1.91 (1.50)	3.23 (2.07)	4.41 (2.25)	4.87 (2.20)	3.71 (2.12)	3.44 (2.14)	5.11 (1.96)
Variable range	19–70	--	0–150	27–134	1–7	1–7	1–7	1–7	1–7	1–7	1–7	1–7

*Notes*.

*** Significant at *p* < .001

** *p* < .01

* *p* < .05. For sex, 1 = male, 2 = female.

Table 3 displays the intercorrelations between demographic variables, specific beliefs about being able to engage in secondary tasks and drive safely, attitudes, intentions, social norms and perceived behavioural control. Age and hours driven were not significantly related to any of these variables. TABP showed weak, positive relationships with the general attitude towards mobile phone use while driving, social norms towards mobile phone use while driving, and sex.

**Table 3:** Associations between age, sex, hours driven per week, TABP, specific beliefs about being able to engage in secondary tasks and drive safely, and TPB variables (N = 220).

While driving, how often do you…	1	2	3	4	5	6	7	8	9	10	11
1. Age	--										
2. Sex	.10	--									
3. Hours driving per week	.01	-.13	--								
4. TABP	.03	-.17*	-.02	--							
5. Belief about being able to drive safely and read a text message	-.08	.10	-.01	-.01	--						
6. Belief about being able to drive safely and write a text message	-.10	.10	.05	-.03	.83**	--					
7. Belief about being able to drive safely and talk on a mobile phone	-.05	.02	.01	.02	.44***	.40***	--				
8. Belief about being able to drive safely and talk with a handsfree unit	.05	-.09	-.01	.05	.21**	.15*	.60***	--			
9. General intention towards mobile phone use while driving	-.01	.02	.09	.10	0.3	.06	.17**	.09	--		
10. General attitude towards mobile phone use while driving	-.02	-.10	.08	.15*	.05	.10	.08	-.02	.68***	--	
11. Social norms towards mobile phone use while driving	-.02	-.02	-.12	.16*	.03	.05	.08	-.03	.60***	.80***	--
12. Perceived behavioural control	-.03	-.05	-.06	.03	-.08	-.11	.05	.05	.18**	.20***	.23***

*Note*.

*** Significant at *p* < .001

** *p* < .01

* *p* < .05.

### Relationships between interactions with mobile phones while driving and perceptions about being able to do this safely

Hierarchical stepwise regressions were conducted on each of the selected interactions with a mobile phone. These were taking “selfie” pictures while driving, typing an address into a map application, reading or writing an email, making a video call and voice call using an application, reading and writing a text message, as well as reading and writing social network posts. In the first block, demographic information such as age, sex, driving purpose, and hours spend driving per week were entered. In the second block, the Bortner (TABP) scale scores were entered. In the third block, specific beliefs about being able to engage in secondary tasks and driving safely were entered. In the final block, the TPB variables such as the general attitude and intention towards phone use while driving, social norms towards mobile phone use and perceived behavioural control were entered. All were entered using the stepwise procedure, the results of which are presented in Table 4.

**Table 4:** Factors related to different types of mobile phone applications use while driving (N = 220).

	B (95% CI)	*β*	*p*
1. Take photos “selfies”	R^2^ 0.03; F (1,218) = 7.03, *p* = 0.009		
Belief about being able to drive safely and write a text message	-.25 (-.44--.07)	-.18	.009
2. Type an address into maps	R^2^ 0.04; F (1,218) = 11.18, *p* = 0.001		
Belief about being able to drive safely and write a text message	-.31 (-.49--.13)	-.22	.001
3.Write an email	R^2^ 0.01; F (1,218) = 4.15, *p* = 0.04		
General attitude towards mobile phone use while driving	-.14 (-.28--.01)	-.14	.043
4.Read an email	R^2^ 0.02; F (1,218) = 4.15, *p* = 0.04		
Belief about being able to drive safely and read a text message	-.19 (-.37--.01)	-.14	.043
5. Make a call using mobile phone applications	R^2^ 0.03; F (1,218) = 7.02, *p* = 0.009		
Belief about being able to drive safely and write a text message	-.18 (-.43--.06)	-.18	.009
6. Read/look at posts on social networks	R^2^ 0.03; F (2,217) = 4.24, *p* = 0.041		
Belief about being able to drive safely and write a text message	-.19 (-.39--.01)	-.13	.06
General attitude towards mobile phone use while driving	-.15 (-.28--.01)	-.14	.04

As can be seen in Table 4, the only factors that were related to mobile phone usage were the beliefs about being able to engage in secondary tasks and drive safely and positive attitudes regarding the use of a mobile phone while driving. The regression analyses for using an intellectual assistant, making a video call, writing posts on social networks, and reading / writing text messages were not significant. With regard to taking “selfies”, typing an address onto a map application and making a call using applications, drivers who believed they were more able to drive safely while writing a text were more likely to engage in these behaviours. In terms of writing an email and reading social network posts, a positive attitude towards the behaviour was the key factor related to increased engagement in these activities. Age, sex and type A behaviour were not significantly associated with any of the mobile phone interactions.

## Discussion

The aim of the current study was to explore the frequencies of mobile phone applications use while driving, as well as to examine the psychosocial factors that influence driver willingness to use these applications despite the legislative bans in Ukraine. As such, we explored the associations between the TPB variables, TABP, as well as specific beliefs about being able to engage in secondary tasks and drive safely and the frequencies of self-reported mobile phone applications usage while driving. Interestingly, no previous studies have attempted to investigate this issue despite the fact that the functionality of mobile phones has rapidly increased over the last 10 years.

In general, our results showed that the Ukrainian drivers are most likely to use mobile phone applications for reading text messages, followed by sending text messages, taking selfies, typing an address into maps and writing / reading an email. The self-reported frequencies overall suggest that participants regularly use mobile phone applications while driving, which consequently may pose a safety risk not just to the drivers themselves, but also to other road users. When exploring the frequencies of standard mobile phone functions usage, scholars have previously reported that drivers most frequently use their mobile phones for answering / making calls, with much smaller proportions of drivers reporting reading / sending text messages while driving (e.g., [22,42]). Our overall findings make an important contribution to the existing knowledge, suggesting that the frequency of mobile phone applications usage should be considered alongside the standard mobile phone functions to identify the different dimensions of this risky behaviour.

Our findings indicated that a large proportion of the participants would read and write a text message using mobile phone applications while driving. Because the process of writing / reading text messages using the existing applications, such as Viber and WhatsApp, is exactly the same as writing / reading using a traditional text messaging function, this finding is particularly concerning (e.g., [13,15]). This is because when drivers are actively engaged in texting behaviour, they look 400 times more away from the road [25,43]. In addition, nearly one third of the drivers read and/or wrote an e-mail at least once while driving in the previous year. This is concerning, given that this secondary task is as demanding on cognitive resources as writing a text message. What remains unknown and requires further research, however, is an understanding of what kind of emails/text messages (e.g., work-related or personal) drivers tend to deal with while driving a vehicle. For example, a study conducted by Porter and Kakabadse [44] on the association between the use of technology and work addiction showed that those individuals who are workaholics routinely use technology outside work hours / environment. When applied to the driving context, this could possibly mean that those drivers who tend to access their work emails / read text messages from outside work hours / environment may also have an urge to do so while driving. Understanding the motivations across different mediums will help design strategies to reduce distraction behaviour.

Nearly one quarter of the participants in our study had made a call using mobile phone applications at least once in the past year. In contrast to this, a previous study by Sullman et al. [22] employing a sample of the Ukrainian drivers showed that approximately 90% of participants reported making or answering a call at least once in the past year using a standard mobile phone function. Given that making a call via a mobile phone application while driving requires a sufficient Wi-Fi network connection, drivers may choose to use a standard phone call function over the applications simply because of the practicality. In addition, making a standard call while driving could be considered a preferable option for drivers in terms of ease of use.

Reading and / or writing posts on social networks were found to be relatively common amongst drivers, although approximately 70% of the sample reported they had never engaged in this behaviour. This finding aligns with research from the UK [45] that found 24% of young adults and 8% of adults admitted to using their mobile phones for social networking while driving. This behaviour is of particular concern, as the results of a driving simulator study conducted jointly by the IAM RoadSmart charity and the Transport Research Laboratory (TRL) indicated that using a mobile phone for social networking increases reaction times by 37.6%, while driving under the influence of cannabis, for instance, increases reaction times by 21% [17]. One possible explanation for this finding could be that those individuals, who overuse social networking applications on mobile phones in a day-to-day life, may not be able to control this behaviour in the driving context. Previous research commonly explains this type of problematic phone use behaviour within the terms of mobile phone dependency (e.g., [31,46]) social media addiction (e.g., [47,48]), and technology addiction (e.g., [49]). In addition, making and / or reading posts on social networks could be used by drivers as a way to deal with their driving experience or to regulate emotion. For instance, Stephens et al. [50] found that across a 13-month period, over 80,000 twitter posts # road rage were made and seemingly while driving. A large amount of these included a 7- second video clip, filmed by the drivers in situ.

Rather alarmingly, almost half of the drivers in our study reported taking a selfie while driving at least once in the last year. However, this finding is somewhat unsurprising as it is estimated that at least one million selfies are taken per day worldwide [51], which highlights how prevalent this phenomenon is these days. In order to explain the reasons why people take selfies in general, previous research has mostly concentrated on finding the associations between this behaviour and various personality traits, such as narcissism (e.g., [52]), attention seeking and low self-esteem (e.g., [53]). In the driving context, drivers may possibly take selfies to reinforce their self-concept given that having a vehicle in Ukraine may still be considered to be prestigious. In addition, as previously suggested by Stephens et al. [50] taking selfies may also play a role in the processing of the driving experience or emotion regulation. Future research should further explore the goals/motivations behind using social networking applications by drivers and how individual differences may impact their use.

The results of this study indicate that both general positive attitudes towards using mobile phone while driving and approval of this behaviour by significant others were significantly related to frequent mobile phone application usage amongst the Ukrainian drivers. This includes sending a text message and making a call using mobile phone applications, reading / looking at posts on social networks, writing and reading an email, as well as typing an address into a map application. The results also showed that a positive attitude towards mobile phone use while driving was related to frequent writing of emails and reading posts on social networks. This is somewhat consistent with previous research, in that drivers who had positive attitudes towards mobile phone use and who perceive that their significant others would approve of this behaviour tended to use standard mobile phone functions more frequently while driving (e.g., [27,54,55]). This finding may indicate that drivers form positive attitudes towards this dangerous behaviour despite being aware of the risk associated with engaging in it while driving. In fact, previous research has shown that most drivers are aware of the risk of using a mobile phone while driving (e.g., [56,57]); however, they still engage in this behaviour as the perceived benefits of it may outweigh the associated risks [58]. In addition, being aware of the risks does not necessarily serve as a disincentive factor when it comes to engaging in this behaviour (e.g., [59]). As for the association between social approval and frequencies of mobile phone usage while driving, this highlights the necessity of incorporating themes of social influence when developing interventions to tackle this dangerous behaviour more efficiently (e.g., [25]).

Our results also showed that those drivers, who had stronger beliefs about being able to drive safely and engage in secondary tasks, used their mobile phones while driving more frequently. This finding was supported by the results of the hierarchical stepwise regressions, indicating that beliefs about being able to drive safely and write or read a text message affect the frequency of using a mobile phone to take a selfie while driving, as well as typing an address onto maps and making a call. This finding can be explained in two ways. Firstly, it suggests that drivers might not always be able to detect and accurately judge changes in their driving performance caused by simultaneously using a mobile phone and driving. In addition, De Craen et al. [60] report that those drivers who inaccurately assess their driving performance and skills tend to engage in driving and secondary tasks that are too cognitively demanding and potentially unsafe. Secondly, most drivers tend to be overconfident in their driving skills, which may subsequently lead to overestimating their multitasking abilities.

Finally, in the current sample of the Ukrainian drivers, type A behaviour pattern did not significantly predict any type of mobile phone applications usage. Although previous research has reported there to be a strong link between the TABP and RTAs (e.g., [33,34]), our study revealed that the reasons drivers use mobile phone applications while driving may only be slightly related to their impatience and time urgency.

### Limitations

This study has several limitations. Firstly, the data were collected using self-report questionnaires, which may have been affected by social desirability bias. However, in order to mitigate its influence, participants were assured of anonymity and confidentially, which is considered an efficient measure against this phenomenon (e.g., [61]). Secondly, the vast majority of the participants were males, which may reduce the ability to generalise these findings to the driving population of Ukraine. However, given that only 22% of Ukrainian drivers are females, according to the Marketing Index TNS Global report [62], the study sample was comparatively representative of the general population, in terms of the sex ratio. In addition, the study participants were relatively young, which means that they might have been more familiar with smart phones applications and therefore would be more likely to use them while driving. Thirdly, although some of the participants were invited to take part in the study using a snowballing technique, a large proportion of the sample were students / staff from the National Aviation University and members of the Ukrainian motorists’ forum. It can be presumed that they may significantly differ from the general population in terms of their socio-economic status, access to technology and types of the mobile phones they use (smart phones versus older mobile phones). Considering these points, future research should aim to examine the psychosocial factors relating to the mobile phone applications use among a more representative sample.

### Summary and practical implications

Despite the growing functionality of mobile phones and continuous introduction of new applications that significantly increase an individuals’ level of mobile phone involvement in day-to-day life, there is little information regarding how these innovations affect the prevalence of mobile phone use while driving. To our knowledge, this study is one of the first to report the frequencies of mobile phone applications use, which was conducted using a sample of Ukrainian drivers. Our results revealed that the majority of drivers would frequently read / write a text message, type an address into maps and write / read an email while driving. Given the known risk associated with writing / reading a text message, the frequency of these behaviours is concerning. In addition, a large proportion of drivers reported haven taken a selfie while driving in the past year; suggesting social media may have infiltrated the driving experience. The engagement with various types of applications may pose additional risks to individuals’ driving performance, thereby potentially increasing the chances of crash involvement. The reported frequencies highlight the need to include mobile phone applications when exploring the “real” levels of mobile phone use when driving. In addition, and in line with previous road safety research, positive attitudes, perceived social approval and lack of risk acceptance were all positively related to mobile phone use. This suggests that future research should further explore the goals/motivations behind using mobile phone applications while driving and how individual differences may impact their use. Lastly, future studies should also explore the perceived risks related to mobile phone applications usage while driving.
